# Well-being and Burnout Amongst Interventional Radiologists in the United Kingdom

**DOI:** 10.1007/s00270-023-03455-5

**Published:** 2023-06-28

**Authors:** Ahmad Al Rekabi, Mitch Chen, Neeral Patel, Robert Morgan, Ian McCafferty, Philip Haslam, Mohamad Hamady

**Affiliations:** 1 Division of Interventional Radiology, Imperial Academic and Healthcare NHS Trust, 53 humes avenue, London, W72LJ UK; 2 Department of Radiology, St George’s NHS Trust, London, UK; 3 Department of Radiology Birmingham, Birmingham, UK; 4 Department of Interventional Radiology, Newcastle Upon Town NHS Trust, Newcastle Upon Tyne, UK; 5 Department of Surgery and Cancer, Imperial College London, London, UK

**Keywords:** Interventional radiology, Burnout, Wellbeing, Workforce, Workload

## Abstract

**Purpose:**

To assess the prevalence of burnout amongst Interventional Radiologists (IRs) in the United Kingdom and identify demographic and practice-related stressors that may adversely affect well-being.

**Materials and Methods:**

A survey of 36 questions was divided into two sections. Section A consisted of 14 questions that assessed demographics and work characteristics; Section B assessed burnout, utilizing the 22-item Maslach burnout inventory. Four additional open-ended questions were included to allow participants to voice opinions on the biggest contributors to workplace burnout and plans that could be implemented to alleviate this. The questionnaire was distributed to the British Society of interventional (BSIR) members. The study was conducted between August and September 2022.

**Results:**

Moderate to severe scores in emotional exhaustion (EE) were recorded in 65% of participants (moderate 26%; severe 39%) of participants r. Moderate to severe depersonalization (DP) scores were recorded in 46% of participants (moderate 23%; severe 23%). Low-moderate levels of personal accomplishment (PA) scores were recorded in 77% of respondents (low 50%; moderate 27%). Weekly hours and out-of-hour IR cover were statistically significant in predicting emotional exhaustion.

Age, sex (male), time available for teaching, and weekly hours were statistically significant in predicting the depersonalisation score. Age was a predictive factor for personal accomplishment. The most recurring themes in open response to major contributors of burnout were shortage of IR clinicians and supporting staff as well as the increasing IR workload.

**Conclusions:**

This survey has demonstrated high prevalence of burnout amongst Interventional Radiologists in UK. Urgent measures are required to tackle the workforce shortage, recognition of IR workload and control IR resources.

## Introduction

Interventional Radiology (IR) is one of the fastest expanding specialties in modern medicine with a growing scope of practice that has a role in almost every area of healthcare. The Royal College of Radiologist (RCR) provisions of interventional radiology report stated the importance of having access to a robust 24/7 IR service in an acute hospital [1]. To achieve this, the RCR suggests that a service should consist of six or more IR consultants, with units covering a population over one million people requiring eight or more consultants. The British Society of Interventional Radiologists (BSIR) in collaboration with Department of Health identified a substantial variation in access to potential lifesaving IR procedure across the UK, ranging from a 22% shortfall in London to a 51% shortfall in the north of England. The 2021 RCR census reported that 98% of clinical directors were worried about workforce morale, stress and burnout in their department [2].

Burnout in the workplace is a cluster of symptoms resulting from chronic work-related stressors that manifest amongst other things as emotional exhaustion, depersonalization and reduced personal accomplishment. Emotional exhaustion is defined as feeling of being overextended and depleted of emotional and physical resources, and depersonalization is defined as a detached response to various aspects of the job, while reduced personal accomplishment is defined as feeling of incompetence and or inadequate achievement and productivity at work.

Burnout was identified to be significantly more likely in physicians (37.9%) when compared to the general public 27.8% [3]. The toll on healthcare is significant with physician burnout associated with an increased risk of depression, substance abuse, and thoughts of suicide [4, 5]. The largest meta-analysis published by the BMJ examining 170 observational studies consisting of 239,246 physicians identified that physicians with burnout were four times more likely to be dissatisfied with their job, three times more likely to have thoughts or intentions to leave/regret their career choice and twice as likely to be involved in patient safety incidents [6].

Prior studies have demonstrated a high prevalence of burnout amongst diagnostic radiologists as well as interventional radiologists in the USA [7, 8]. However, likely secondary to the varying nature of the job, the complex personal and organization factors contributing to career-related stress, studies have found differing rates of burnout among physicians in different specialties [3]. Similarly, these differences may have similar impact between the two healthcare systems in the USA and United Kingdom. Since identifying demographic and practice patterns associated with burnout is important when devising strategies to reduce burnout in IR, this study aimed to assess the prevalence of burnout amongst interventional radiologist in the United Kingdom and identify demographic and practice-related stressors that may account for this.

## Methods

The survey was composed of a 36-question survey divided into two sections. Section A consisted of 14 questions that assessed demographics and work characteristics; section B assessed burnout, utilized the 22-item Maslach burnout inventory, which is considered the gold standard for evaluating burnout in medical studies. Four additional open-ended questions with free responses were also included at the end, to allow participants to voice their opinion on what they believed were the biggest contributors to workplace burnout and plans that they felt could be implemented to alleviate this.

The study period extended for 4 weeks (August–September 2022) and was distributed to the BSIR members. Email communication contained the link to the survey which was hosted on SurveyMonkey. All responses were anonymous with the website recording the IP address, therefore limiting participants to a single response. No compensation was offered to participants for completing the survey and reminder emails were sent periodically during the study phase. No requests to complete the survey were placed on social media or public forums.

### Section A

The demographic section was composed of 14 questions looking at the current stage of practice, age, gender, type of practice (tertiary vs district general hospital), size of practice, multiple site cover, number of hours worked and on-call commitment.

### Section B: Maslach Burnout Inventory-Human services Survey (MBI-HSS)

The MBI-HSS consisted of 22 questions assessing three separate domains: emotional exhaustion (EE), depersonalisation (DP) and personal accomplishment (PA). Each domain accumulated a combined score dependent on the severity of symptoms assessed on a seven-point Likert scale ranging from 0 points for “never” and 6 points for “everyday”. Responses for each domain were categorized into low, moderate, and severe; EE, low: 18, moderate 19–26, and severe ≥ 27; DP, low < 5, moderate 6–9, and severe ≥ 10; PA, low ≤ 33, moderate 34–39, and severe > 40. Higher scores on emotional exhaustion and depersonalisation indicated a higher degree of burnout, with the converse being the case for personal achievement. In the design of the MBI-HSS, burnout was defined not as a dichotomous variable (burnout vs no burnout) but instead as a continuous variable with scores ranging from low to high. The list of questionnaire items for section A (demographics) and their descriptions is given in Table 1. Section B, the MBI-HSS questionnaire, is copyright protected but can be obtained from mind garden website [9].

**Table 1:** Description of Section A Questionnaire Items

Item	Description
Seniority	Which best describes your current level of practice in IR?
Age	What is your age?
Sex	What is your gender?
Hospital	Which best describes your type of clinical practice/hospital?
MultiSite	As part of your practice are you required to cover multiple sites?
NoIR	How many interventional radiologists are in your department?
NoIRSess	On average, how many Interventional radiology sessions (programmed activities) do you carry out per week?
NoDSess	How many diagnostic/reporting sessions (programmed activities) do you carry out per week?
TimeTeaching	Do you feel you have sufficient time and energy to engage in teaching/training and/or research and development?
WeeklyHours	On average, how many hours do you work per week?
OOHIRCover	Are you required to cover out-of-hours interventional radiology work?
CallFreq	On average, what is your on-call frequency?
CallPhone	When on-call, how often do you receive calls/have to give clinical advice over the phone?
OOHGoIn	When on-call, how often do you have to go in to work for an out-of-hours case?
EE	Emotional exhaustion
DE	Depersonalisation
PA	Personal accomplishment

To understand the most problematic areas of work life, participants were asked to elaborate on what they believed was the strongest contributors to burnout, if changes had been implanted already in their practice to tackle these factors and what these were, and finally what suggest intervention they believed could tackle burnout in the workplace.

For the purposes of comparison to prior studies, burnout was defined as being present in participants based on the established convention of having at least one of the following on the MBI-HSS; (1) EE score of ≥ 27 and/or (2) DP score of ≥ 10. This convention is the predominant means of identifying burnout based on previously published studies [10–14].

### Statistical Analysis

Descriptive statistics were calculated such that mean (SD) was used for normally distributed variables and median (range) for nonparametric data. Multivariable linear regression analysis was used to identify the confounders between Maslach Burnout Inventory and demographic factors. The 14 potential contributing risk-factors included practice level, age, gender, practice type (District general hospital vs tertiary care hospitals), multiple site cover, size of practice, number of IR programmed session per week, number of diagnostic/reporting programmed session per week, time provided for teaching/clinical research, number of hours worked per week, out-of-hours on-call requirement and frequency of on-calls.

Multivariable linear regression was performed to evaluate correlations between demographic variables and MBI subscale scores (EE, DP and PA). On the interpretation of correlations, negative correlations between MBI scores and demographic factors correlated with compounding factor for increased burnout. Conversely, positive correlations indicated protective factors against burnout. Statistical differences between the groups were assessed using the analysis of variance (ANOVA) test. Multivariable logistic regression was performed to assess the correlations between demographic variables and the “burnt out” status of the participants. *P* values of less than 0.05 were considered statistically significant. Analyses were performed using R 4.2.2 (R Foundation for Statistical Computing, Vienna, Austria).

## Results

### Demographics

The survey hosted on SurveyMonkey was distributed to BSIR members by email, which contains around 800 registered members consisting of consultants and trainees. The survey generated 251 responses (response rate of 31%), with 223 completed questionnaires (28%) over the course of 4 weeks. Interventional radiologist who were not members of BSIR were excluded from the study due to lack of alternative verified database with email correspondence. Only completed questionnaires were included in the study. The results of the demographics from section A including type of practice, working hours and on-call frequency are summarized in Table 2.

**Table 2:** Summary of participants demographics

*Practice level*	
Fellow	27 (12%)
Consultant(< 5 Years)	50 (23%)
Consultant (6–10 years)	36 (16%)
Consultant (> 15 years)	110 (49%)
*Age*	
30–40	72(32%)
40–50	77(35%)
50–60	59(26%)
> 60	15(7%)
*Gender*	
Male	193 (87%)
Female	30 (13%)
*Practice type*	
DGH	66 (30%)
Tertiary hospital	157 (70%)
*Multiple site cover*	
Yes	138 (62%)
No	85 (38%)
*Hours worked per week*	
< 30	3 (1%)
30–40	16 (7%)
40–50	131 (59%)
50–60	50 (23%)
> 60	23 (10%)
*On-call frequency*	
1:2	1 (< 1%)
1:3	6 (3%)
1:4	6 (3%)
1:5	47 (21%)
> 1:5	137 (61%)
No on-calls	26 (12%)
*When on-call, how often do you have to go in to work for an out-of-hours case?*	
Never	3 (1%)
Rarely	12 (5%)
Occasionally	74 (33%)
Frequently	89 (40%)
Very frequently	19 (9%)
No on-calls	26 (12%)
*Number of Interventional radiologists in your department?*	
0–5	77 (35%)
5–10	112 (50%)
11–15	29 (13%)
16–20	5 (2%)
*On average, how many Interventional radiology sessions (programmed activities) do you carry out per week?*	
1–3	41 (18%)
4–6	136 (61%)
7–9	39 (18%)
10–12	7 (3%)
*Do you feel you have sufficient time and energy to engage in teaching/training and/or research and development?*	
Yes	58 (26%)
No	165 (74%)

### Analysis

The majority of participants 165 (74%) reported that they did not feel that they had sufficient time and energy to engage teaching/training, research or development.

#### Burnout Score

**Fig. 1:**
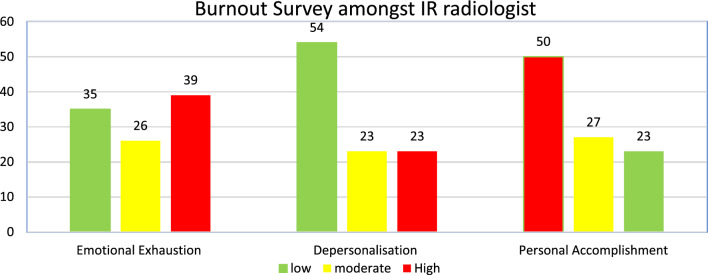
Burnout survey results

Based on the MBI scale, moderate-severe scores in emotional exhaustion (EE) were recorded in 144 (65%) participants, with severe EE recorded in 86 (39%) participants. Moderate-severe scores in depersonalization were recorded in 101 (46%) participants, with severe DP in 51 (23%) participants. Low-moderate levels of personal accomplishment (PA) were recorded in 172 (77%) participants, with low levels of PA recorded in 112 (50%) participants (Fig. 1).

Mean ± SD scores for EE, DP and PA were 24 (± 12), 6 (± 6) and 33 (± 8), respectively. Median values were 23, 5, and 33, respectively.

Based on the previously established definition of burnout, 97 (44%) participants met one or more of the criteria for burnout.

#### Demographic Factors

**Fig. 2:**
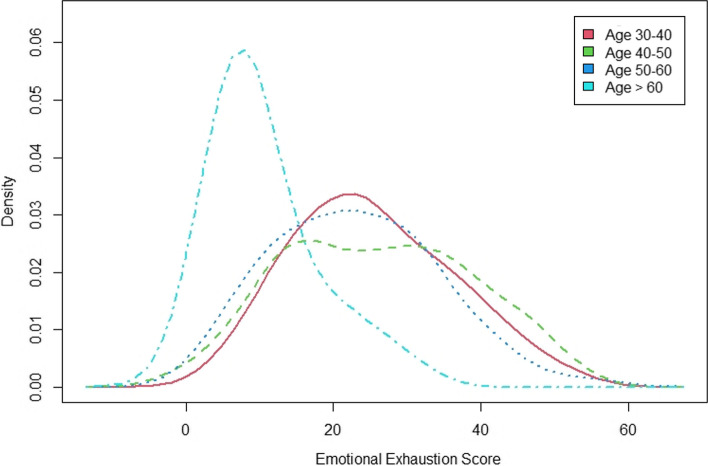
EE score stratified by age groups

**Fig. 3:**
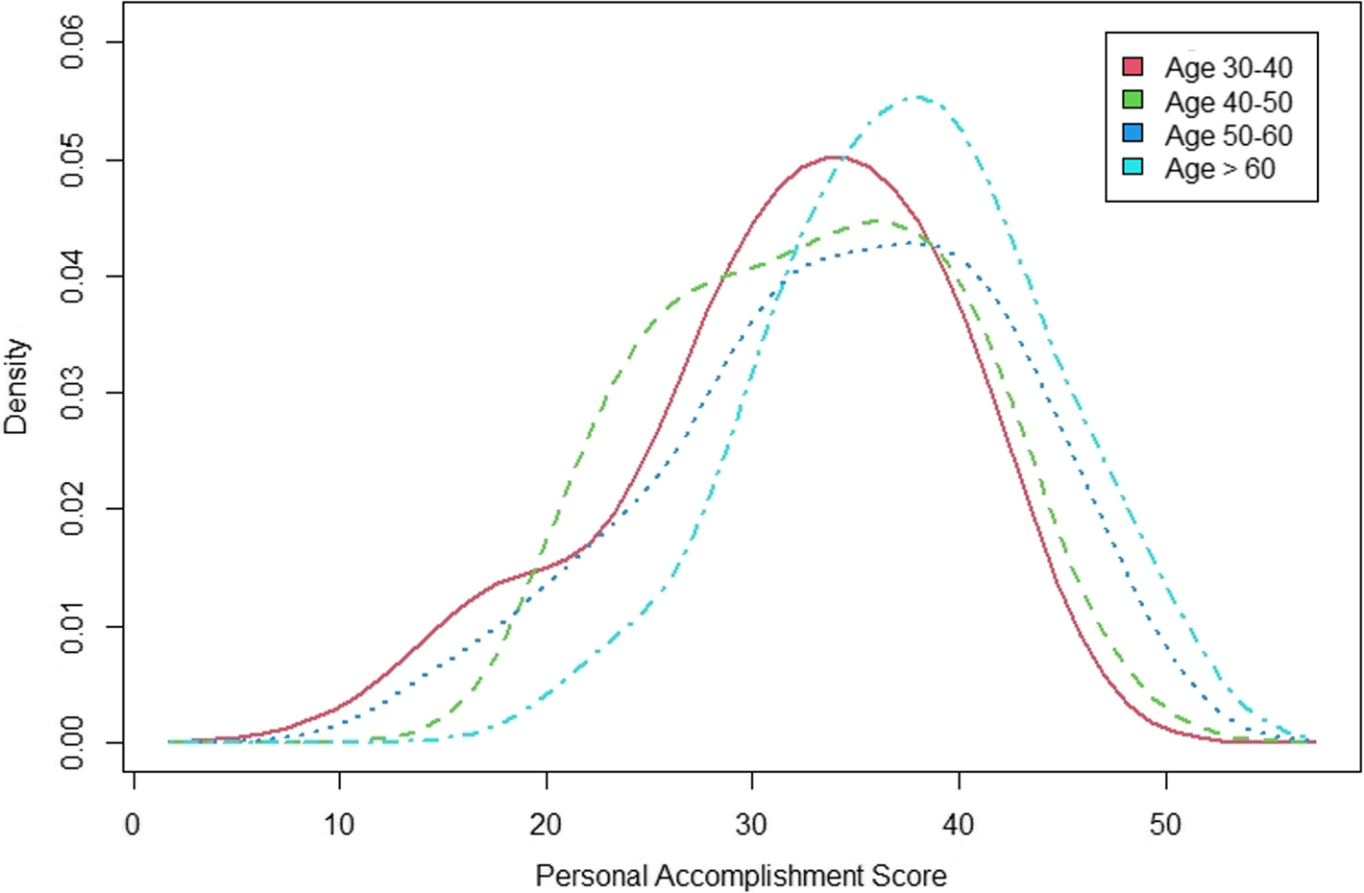
PA score stratified by age groups

**Fig. 4:**
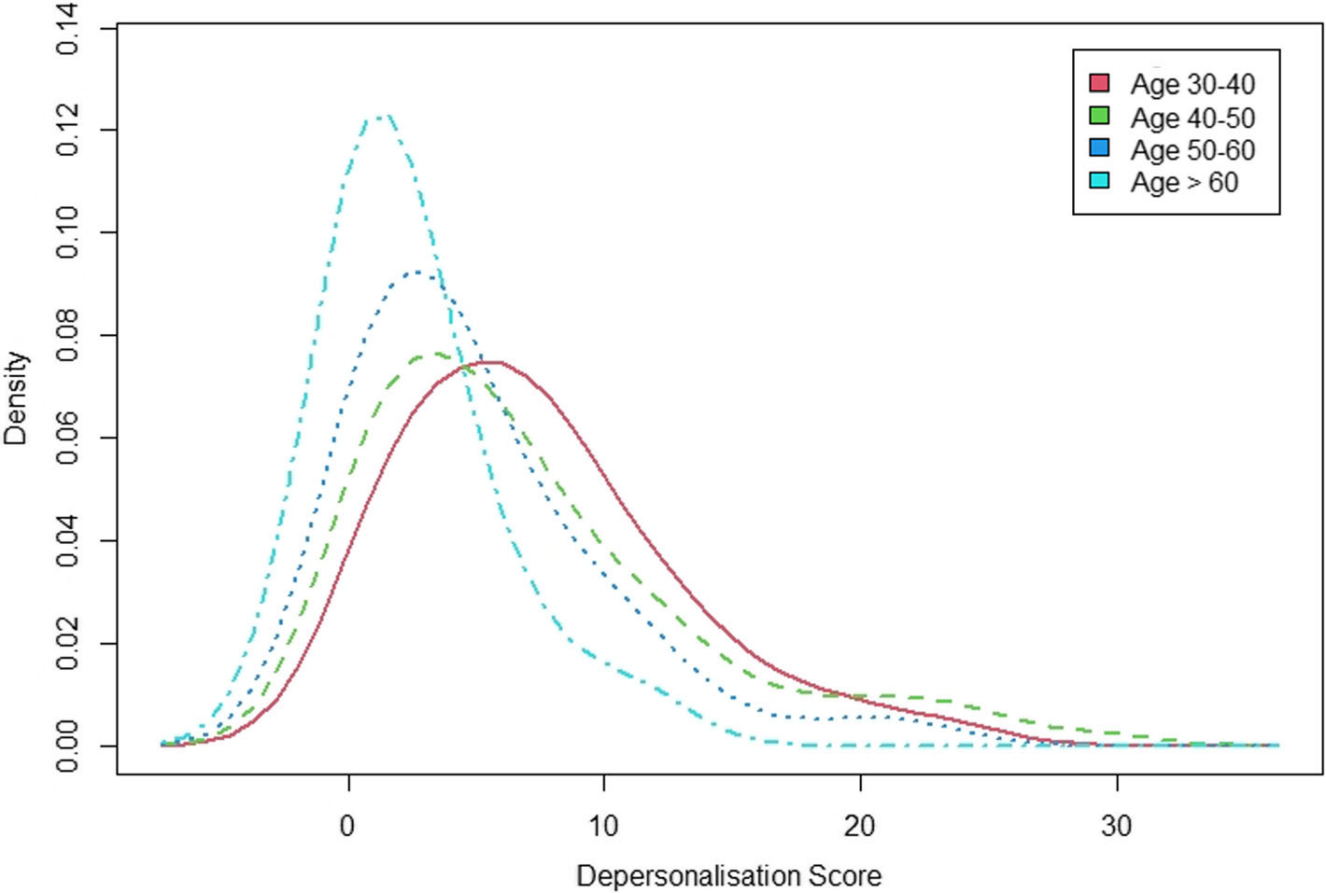
DP score stratified by age groups

Stratified by participants’ age group, those older than 60 reported the lowest emotional exhaustion score and the remaining groups reported similar scores (*p* ≤ 0.05). The depersonalisation score decreased from the youngest to oldest participant age group (*p* ≤ 0.05). Personal accomplishment scores showed a reverse trend, with the oldest participants reporting the highest scores (*p* ≤ 0.05) (Figs. 2, 3, 4).

Linear regression analyses showed that age, seniority, time available for teaching, weekly hours and out-of-hour IR cover were found to be statistically significant in predicting the emotional exhaustion of the participants (Table 3); age, sex, time available for teaching, and weekly hours (highlighted in bold) were predictive the depersonalisation score (Table 4); and only age was predictive of personal accomplishment score (Table 5).

**Table 3:** Emotional exhaustion regression findings

Predictors	Emotional exhaustion		
	Estimates	CI	*p*
(Intercept)	25.53	15.00–36.05	** < 0.001**
Seniority	2.84	0.64–5.05	**0.012**
Age	− 5.62	− 8.24 to − 2.99	** < 0.001**
Sex [2]	− 2.61	− 6.67–1.45	0.207
Hospital [Teaching tertiary hospital]	1.81	− 1.50–5.11	0.284
MultiSite [1]	− 0.45	− 3.37–2.47	0.763
NoIRSess	0.24	− 0.67–1.15	0.606
NoDSess	− 0.58	− 1.49–0.32	0.208
TimeTeaching [1]	− 6.92	− 10.19 to − 3.64	** < 0.001**
WeeklyHours	2.50	0.65–4.35	**0.008**
OOHIRCover [1]	− 8.04	− 14.37 to − 1.72	**0.013**
CallFreq	− 0.33	− 1.88–1.22	0.677
CallPhone	0.78	− 1.29–2.86	0.459
OOHGoIn	1.69	− 0.43–3.81	0.118
Observations	223		
*R*^2^	0.283		

Age, seniority, time available for teaching, weekly hours and out-of-hour IR cover (highlighted in bold) were found to be statistically significant in predicting the emotional exhaustion of the participants

**Table 4:** Depersonalisation score regression findings

Predictors	Depersonalisation		
	Estimates	CI	*p*
(Intercept)	9.43	3.90–14.95	**0.001**
Seniority	0.91	− 0.26–2.07	0.126
Age	− 2.58	− 3.94 to − 1.22	** < 0.001**
Sex [2]	− 2.37	− 4.47 to − 0.26	**0.028**
Hospital [Teaching tertiary hospital]	1.73	− 0.22–3.68	0.082
MultiSite [1]	0.87	− 0.67–2.41	0.268
NoIR	− 0.17	− 0.43–0.09	0.192
NoIRSess	− 0.09	− 0.56–0.38	0.720
NoDSess	0.10	− 0.37–0.57	0.677
TimeTeaching [1]	− 2.41	− 4.10 to − 0.72	**0.005**
WeeklyHours	0.97	0.01–1.93	**0.047**
OOHIRCover [1]	− 2.49	− 5.76–0.78	0.136
CallFreq	0.15	− 0.65–0.95	0.720
CallPhone	− 0.08	− 1.16–0.99	0.878
OOHGoIn	0.40	− 0.70–1.49	0.476
Observations	223		
*R*^2^	0.183		

Age, sex, time available for teaching, and weekly hours (highlighted in bold) were found to be statistically significant in predicting the depersonalisation score of the participants

**Table 5:** Personal accomplishment score regression findings

Predictors	Personal accomplishment		
	Estimates	CI	*p*
(Intercept)	30.66	23.00–38.33	** < 0.001**
Seniority	− 1.15	− 2.77–0.46	0.161
Age	2.74	0.86–4.63	**0.004**
Sex [2]	1.79	− 1.14–4.71	0.231
Hospital [Teaching tertiary hospital]	− 1.08	− 3.78–1.62	0.433
MultiSite [1]	− 0.92	− 3.05–1.22	0.400
NoIR	− 0.06	− 0.42–0.29	0.725
NoIRSess	− 0.14	− 0.79–0.51	0.679
NoDSess	− 0.39	− 1.04–0.26	0.236
TimeTeaching [1]	2.25	− 0.10–4.60	0.060
WeeklyHours	− 0.07	− 1.40–1.26	0.919
OOHIRCover [1]	1.69	− 2.84–6.22	0.464
CallFreq	− 0.14	− 1.25–0.97	0.806
CallPhone	0.38	− 1.10–1.87	0.614
OOHGoIn	− 0.61	− 2.13–0.91	0.432
Observations	223		
*R*^2^	0.096		

Only age (highlighted in bold) was found to be statistically significant in predicting the personal accomplishment score of the participants

In a multivariable logistic regression predictive model for burnout, age, seniority and time available for teaching were found to be statistically significant in predicting the outcome (Table 6).

**Table 6:** Burnout logistic regression findings

Predictors	Burnout		
	Odds ratios	CI	*p*
(Intercept)	3.44	0.32–39.11	0.310
Seniority	1.89	1.16–3.13	**0.012**
Age	0.36	0.19–0.64	**0.001**
Sex [2]	0.77	0.32–1.78	0.539
Hospital [Teaching tertiary hospital]	1.23	0.55–2.75	0.613
MultiSite [1]	0.87	0.46–1.67	0.683
NoIR	1.01	0.91–1.13	0.782
NoIRSess	0.94	0.77–1.14	0.511
NoDSess	0.89	0.71–1.09	0.262
TimeTeaching [1]	0.25	0.11–0.53	** < 0.001**
WeeklyHours	1.32	0.89–1.97	0.169
OOHIRCover [1]	0.29	0.07–1.13	0.080
CallFreq	0.96	0.69–1.34	0.827
CallPhone	1.02	0.66–1.57	0.944
OOHGoIn	1.26	0.80–1.99	0.312
Observations	223		
*R*^2^			

Age, seniority and time available for teaching (highlighted in bold) were found to be statistically significant in predicting the burnout status of the participants

### Open-Ended Questions

Four optional questions were included at the end of the survey, two multiple choice question and two open response questions (Table 7).

**Table 7:** Open-ended questions

Question	Answers
(1) What do you believe are the strongest contributors to burnout in your IR department? (Select one or more options)	Diagnostic workload
	IR workload
	Shortages of IR clinicians
	Shortages of IR ancillary staff
	Frequency of on-calls
	Extra clinical duties
(2) Are there any changes that have been made in your IR department to combat burnout?	Yes
	No
(3) If yes, please provide a short summary of those changes?	Open response
(4) Are there any strategies you would like to see implemented to tackle burnout amongst IRs? (Locally or nationally)	Open response

**Fig. 5:**
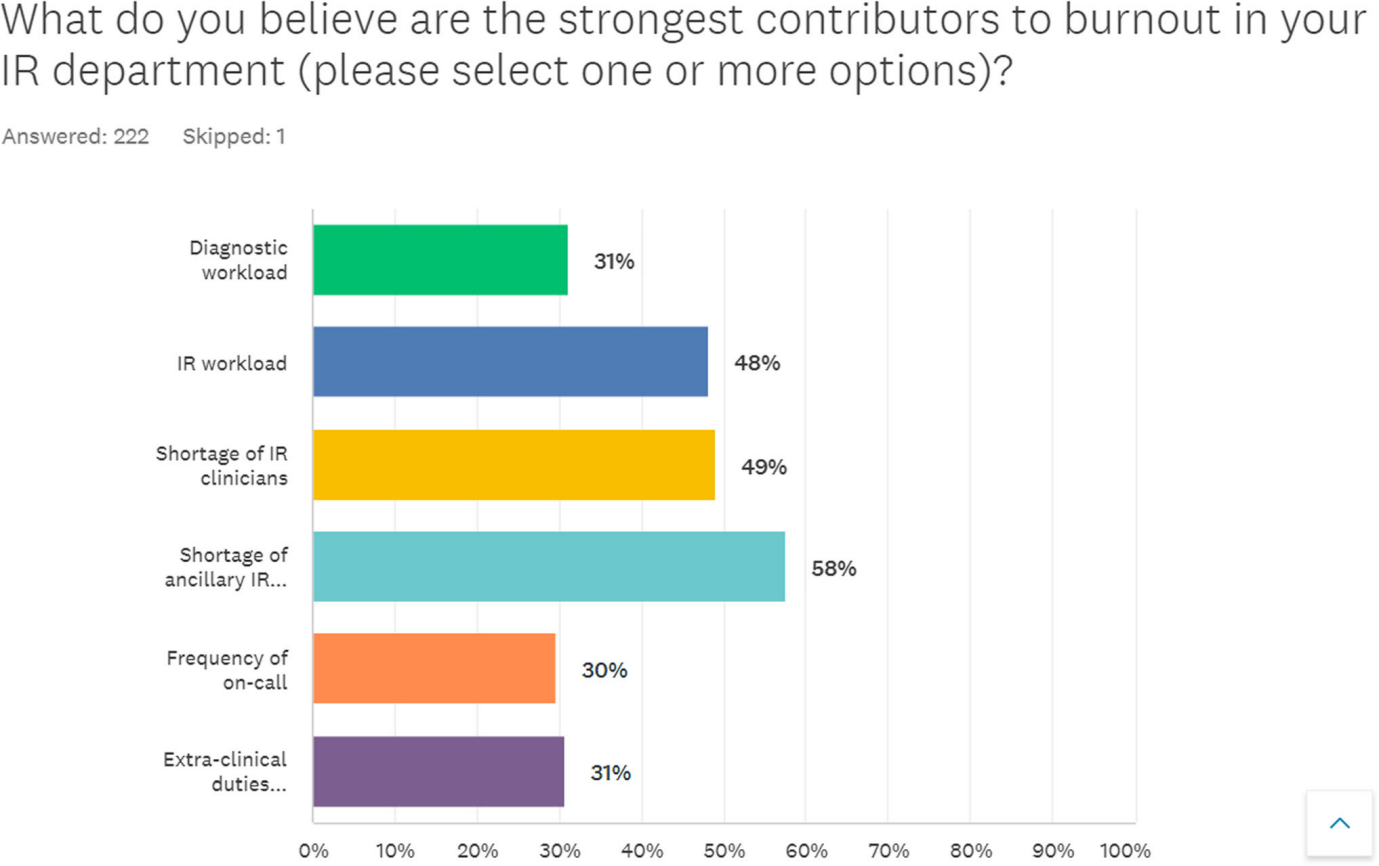
IR clinicians’ opinion on the strongest factors contributing to burnout in the workplace

A total of 222 respondents (99.5%) completed the first question. The most selected option by respondents was “shortage of ancillary IR staff” 128 (58%), followed by “shortage of IR clinicians” 109(49%) closely followed by “IR workload” 107 (48%) (Fig. 5).

A total of 223 respondents (100%) completed the second question, with 178 (80%) answering no and 45 (20%) answering yes. Of the 45 respondents who answered yes, 44 (98%) completed the third question. On review of the responses, the recurring themes included increased recruitment of new IR consultants, improvement in frequency of on-call rota by combining workload with adjacent trusts, providing time off after overnight on-call and provisions of home reporting for diagnostic sessions.

A total of 124 respondents (56%) completed the final question. On review of the responses, there were several recurrent themes that emerged from the answers. These included increased recruitment of IR clinical staff, increased retention of IR ancillary staff with improved career prospects and progression, healthier balance between interventional and diagnostic workload, improved recognition of IR services and workload, and providing dedicated time and resources for IR trainees and consultants.

## Discussion

This survey assessed levels of burnout and well-being amongst UK IRs. The study received 196 responses from consultant interventional radiologists representing over a quarter of the estimated 728 interventional radiologists working in the UK [2]. The results demonstrated 65% of participants reported a moderate to severe degree of emotional exhaustion and 77% of participants reported low-moderate levels of personal achievement. Despite these adverse factors, 54% of participants demonstrated low levels of depersonalization, maintaining a strong degree of empathy and compassion with their patients. The findings in this survey demonstrate similar trends to the survey of interventional radiologist in the USA in the domain of emotional exhaustion and personal achievement. However, the depersonalization domain appears to be less severe amongst the interventional radiologist in the United Kingdom. This could be related to the differences in health practice culture which including demand, financial motivation and working hours.

The study indicated that the level of burnout amongst IRs was nearly double the rates reported in the general public (28%), with 44% presenting with at least one or more manifestations of burnout [8]. This represents a worrying statistic, given clinicians with burnout have been found to be twice as likely to be involved in a patient safety incidence [6]. A study by Shanafelt et al. [15] which focused on American surgeons revealed that for every single-point increase in DP and EE scores, there was an associated 11% and 5% increase in reporting a medical error, respectively. Furthermore, a study of general surgeons by Balch et al. [16] revealed an increased likelihood of self-reported burnout in surgeons involved in a malpractice lawsuit in previous two years, compared with physicians who were not.

Identifying factors associated with high rates of burnout is important and could facilitate the implementation of strategies to combat burnout. This study revealed factors associated with increasing scores on the EE and DP subscale. Clinicians who had been practicing as consultants for a longer period of time were more likely to report higher scores of EE and DP. On average, the different age groups demonstrated similar degrees of EE and DP aside from those over 60 years, who were associated with lower EE and DP scores. This is in line with a similar previous study which found that middle-career physicians were more likely to manifest burnout than early or late-career physicians [17].

Higher scores on the EE subscale were associated with clinicians who provided out-of-hour cover. Furthermore, there was a positive correlation between the number of hours worked per week and a high EE score. The association between the increasing number of working hours and the likelihood of burnout have been highlighted in several prior studies, with prolonged working hours identified as the third leading cause of burnout among physicians [18–20]. Clinicians who felt they did not have enough time and energy for teaching or research were also associated with a higher score on the EE scale.

Analysis of the factors associated with higher DP scores revealed similar results. Younger clinicians, those working more hours and those who answered “No” to the question “Do you have enough time and energy for teaching or research”, exhibited higher depersonalization scores. Male IRs were associated with higher depersonalization scores compared to their female counterparts. This is in contrast to a prior study of burnout amongst IRs in the USA, which found that female clinicians were at a significantly higher risk of burnout [8].

The study demonstrated a positive correlation between the age of participants and personal accomplishment scores. Clinicians who had been practicing for a longer time might have a greater feeling of personal accomplishment in what they have achieved in their career in comparison with their younger counterparts.

In line with factors influencing scores on the burnout subscale, the factors that were significant in predicting the burnout status of the participants were age, seniority, and time available for teaching. The results of the current study in addition to the responses to the open-ended questions indicate the overwhelming factors leading to burnout are the increasing workload, increasing on-call demands, an insufficient workforce, and the lack of resources to meet those demands.

Previous studies which have attempted to examine strategies to reduce physician burnout have primarily focused on improving resilience amongst clinicians and enhanceability to handle stress. A randomized control trial that examined the impact of a biweekly facilitated discussion group in 74 physicians led to a sustained 15.5% reduction of scores on the depersonalization scale [21]. Although physician wellness programmes may tackle one aspect of burnout, organizational factors such as workforce shortage and increasing workload need to be systematically addressed to achieve real change.

The nature of this voluntary questionnaire study lends itself to several limitations. Firstly, there is a degree of selection bias, as respondents with a manifestation of burnout may be more inclined to participate in the survey to voice their dissatisfaction. Equally, it could be argued that clinicians suffering burnout would be less likely to engage in a voluntary survey. A second limitation of this study is the method of distribution. Interventional radiologists who are not members of BSIR would not have the opportunity to participate in the survey. However, if the survey had been distributed on social media to reach a wider audience, it would have been difficult to maintain the validity of the survey and to ensure its completion by only IRs.

## Conclusion

In conclusion, this survey of the United Kingdom (UK) interventional radiologists demonstrated a self-reported burnout prevalence of 44%, almost double that reported in the general public. This is similar to the national average amongst physicians across other specialties and we hypothesize this trend is likely to be similar across most European countries [3].

This represents a concerning figure, given the negative impact that burnout has on job satisfaction, staff retention, career longevity, mental health, and patient care [6]. Urgent measures need to be implemented to tackle the workforce crises, retention of Interventional radiology (IR) clinicians and supporting staff, recognition of IR workload amongst healthcare authorities and the need for greater managerial control over IR resources.
